# Oxygen concentration affects de novo DNA methylation and transcription in in vitro cultured oocytes

**DOI:** 10.1186/s13148-021-01116-3

**Published:** 2021-06-28

**Authors:** Florence Naillat, Heba Saadeh, Joanna Nowacka-Woszuk, Lenka Gahurova, Fatima Santos, Shin-ichi Tomizawa, Gavin Kelsey

**Affiliations:** 1 Epigenetics Program, Babraham Institute, Cambridge, CB22 3AT UK; 2 Diseases Network Research Unit, Faculty of Biochemistry and Molecular Medicine, Oulu University, Oulu, Finland; 3 Department of Computer Science, King Abdullah II School of Information Technology, The University of Jordan, Amman, Jordan; 4 Department of Genetics and Animal Breeding, Poznan University of Life Sciences, Poznan, Poland; 5 Laboratory of Early Mammalian Development, Department of Molecular Biology and Genetics, University of South Bohemia, 37005 České Budějovice, Czech Republic; 6 School of Medicine, Yokohama City University, Yokohama, Japan; 7 Centre for Trophoblast Research, University of Cambridge, Cambridge, CB2 3EG UK

**Keywords:** Oocyte, Mouse, DNA methylation, Transcription, In vitro culture, Normoxia, 5% oxygen

## Abstract

**Background:**

Reproductive biology methods rely on in vitro follicle cultures from mature follicles obtained by hormonal stimulation for generating metaphase II oocytes to be fertilised and developed into a healthy embryo. Such techniques are used routinely in both rodent and human species. DNA methylation is a dynamic process that plays a role in epigenetic regulation of gametogenesis and development. In mammalian oocytes, DNA methylation establishment regulates gene expression in the embryos. This regulation is particularly important for a class of genes, imprinted genes, whose expression patterns are crucial for the next generation. The aim of this work was to establish an in vitro culture system for immature mouse oocytes that will allow manipulation of specific factors for a deeper analysis of regulatory mechanisms for establishing transcription regulation-associated methylation patterns.

**Results:**

An in vitro culture system was developed from immature mouse oocytes that were grown to germinal vesicles (GV) under two different conditions: normoxia (20% oxygen, 20% O_2_) and hypoxia (5% oxygen, 5% O_2_). The cultured oocytes were sorted based on their sizes. Reduced representative bisulphite sequencing (RRBS) and RNA-seq libraries were generated from cultured and compared to in vivo-grown oocytes. In the in vitro cultured oocytes, global and CpG-island (CGI) methylation increased gradually along with oocyte growth, and methylation of the imprinted genes was similar to in vivo-grown oocytes. Transcriptomes of the oocytes grown in normoxia revealed chromatin reorganisation and enriched expression of female reproductive genes, whereas in the 5% O_2_ condition, transcripts were biased towards cellular stress responses. To further confirm the results, we developed a functional assay based on our model for characterising oocyte methylation using drugs that reduce methylation and transcription. When histone methylation and transcription processes were reduced, DNA methylation at CGIs from gene bodies of grown oocytes presented a lower methylation profile.

**Conclusions:**

Our observations reveal changes in DNA methylation and transcripts between oocytes cultured in vitro with different oxygen concentrations and in vivo-grown murine oocytes. Oocytes grown under 20% O_2_ had a higher correlation with in vivo oocytes for DNA methylation and transcription demonstrating that higher oxygen concentration is beneficial for the oocyte maturation in ex vivo culture condition. Our results shed light on epigenetic mechanisms for the development of oocytes from an immature to GV oocyte in an in vitro culture model.

**Supplementary Information:**

The online version contains supplementary material available at 10.1186/s13148-021-01116-3.

## Introduction

Oocytes contain not only the genetic information, but also the epigenome, which is dynamically changing during oocyte growth, and both need to be transmitted correctly to the next generation. De novo DNA methylation starts at primary follicles and finishes at metaphase II oocytes (M2) [1, 2] to produce functional, mature oocytes which are a prerequisite for proper embryonic development [3].

DNA is methylated at CpG-dinucleotides, and differentially methylated CpGs form domain on genomic DNA which can be either hyper- or hypomethylated. In the M2 oocytes, methylation establishment has been associated with transcription units delimiting these hyper- and hypomethylated domains [3, 4]. As well, groups of CpGs define CpG islands (CGIs) which are found throughout the genome. Most of the CGIs are unmethylated but in specific cell type; for example, in the germ cells, these CGIs are characterised by either low or high methylation levels. Some of these CGIs are associated with imprinted germ line differentially methylated regions (igDMRs). Accurate methylation of igDMRs is crucial for the proper embryonic development and for mono-allelic gene expression during development. Depending on the sex of the gamete, methylation of the igDMRs will follow an either paternal or maternal pattern [5]. When DNA methylation fails to be established or maintained on the igDMRs, consequences may be severe for the embryos; embryonic defects are often seen as a result [6].

Assisted reproduction technology (ART) is a common technique in human infertility treatments. It is based on retrieving late stage M2 oocytes, which are fertilised with sperm in a culture dish to generate an embryo. It is important to understand the mechanisms regulating oocyte methylation using in vitro models. There is evidence that in in vitro*,* culture conditions, such as oxygen concentration, pH, temperature, and medium composition, have an effect on oocyte epigenome, but this connection has not yet been carefully analysed [7]. The oxygen concentration in the ovaries varies from 2 to 8% depending on the species [8], and ART techniques are either carried out at 5% oxygen or 20% oxygen [9]. Studies of methylation of selected imprinted genes in children born from ART have been inconclusive. The data either suggest a change in methylation at imprinted genes which is correlated with a higher risk in developing imprinted disorders, or no correlation is observed between the methylation of imprinted loci and imprinted diseases [10–17]. Similar controversial results have been published in relation to super-ovulation. Super-ovulated oocytes from mice and humans show that *Peg1* and *H19* imprinted genes were differentially methylated in super-ovulated oocytes compared to normally ovulating females, suggesting a link between methylation of imprinted genes and super-ovulation [18], whereas other studies reveal no effect on the methylation of the imprinted genes by super-ovulation [19, 20].

Oocytes are an ideal system to study de novo methylation establishment and its relation to transcription, because replication does not occur in these cells. Recent studies have generated mouse oocyte methylomes to understand the mechanisms behind de novo methylation establishment [3, 21, 22]. The relation between methylation and transcription starts to be unveiled in mouse oocytes [4] highlighting a correlation between gene bodies hypermethylation and transcription [3, 4]. However, less compact DNA associated with low amount of chromatin remodellers will establish methylation first independent of transcription [23]. Dnmt3a with its co-factor Dnmt3L and Dnmt1 methyltransferases establish methylation in oocytes [22]; however, the methylation events are also regulated by histone post-translational modifications. To understand further the interactions between histone post-translational modifications, de novo methylation, and oocyte transcription factors, an in vitro oocyte culture model needs to be developed. This model will allow manipulation of such factors for following their implications during oocyte growth, and such model should operate as early as possible, in primary follicles.

The main aim was to develop an in vitro culture model for molecular studies where epigenetic mechanisms will be analysed to identify and understand their intricate regulations. Towards this goal, first a murine in vitro follicle culture model needed to be optimised for developing immature mouse oocytes (primary oocytes) to germinal vesicle oocytes (GV). Second by manipulating the culture conditions, here two different oxygen concentrations, at normoxia (20% O_2_), a higher oxygen concentration than the physiological one, and 5% oxygen (5% O_2_), closer to the ovarian oxygen concentration, we analysed whether methylation and transcription are correctly preserved in in vitro-grown oocytes as in vivo oocytes. Our data describe for the first time how oxygen levels affect oocyte growth specifically when the oocyte culture starts from follicles containing immature oocytes.

## Results

### Optimisation of follicle-free oocyte culture model

**Fig. 1:**
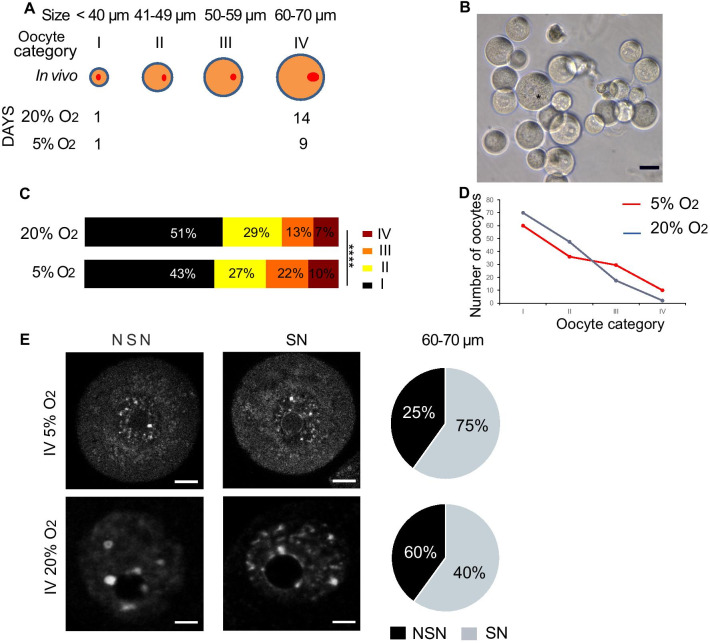
Characterisation of follicle-free cultured oocytes. **a** Schematic representation of the in vitro oocyte culture model. The time of collection of cultured oocytes under 20% O_2_ was day 21 (14 days after start of the culture), and for the oocytes grown under 5% O_2_, it was on day 16 (9 days after the starting the culture). **b** Bright-field photomicrograph of in vitro-grown oocyte cultured for 9 days under 5% O_2_; *indicates oocyte of 70 µm diameter, scale bar 35 µm. **c** Graph of the proportions of oocytes of the various size categories after 14 days of culture (20% O_2_) and 9 days of culture (5% O_2_). Combined data from 7 independent cultures. ****p < 0.001 (ANOVA test). **d** Contingency graph summarising the growth of the cultured oocytes under 20% O_2_ and 5% O_2_. Combined data from 7 independent cultures. **e** DAPI staining of GV-like oocytes grown in 20% O_2_ and 5% O_2_. Pie charts summarising the number of IV oocytes grown under 20% O_2_ and 5% O_2_ classified as non-surrounding nucleus (NSN) and surrounding nucleus (SN) (3 independent oocyte cultures for both conditions, total of oocytes *n* = 68 for 5% O_2_ and *n* = 62 for 20% O_2_, and *n* = 31 GV oocytes aged of 23–25 days old). Scale bar 20 µm

To set up an oocyte in vitro model from immature to germinal-vesicle (GV) oocytes and to be able to manipulate oocytes sufficiently early during their development and maturation, we adopted the protocol from Honda et al. [24], which took advantage of maturing arrested primordial oocytes from dissociated ovaries of 1-week-old mice. In this method, oocytes are allowed to grow in follicle-free cultures, but initially supported by theca-like cells in the primary culture, and we considered that this method would have advantages in allowing manipulations that would be difficult to achieve in follicle cultures. Although oocytes grown by this method have been assessed as completing meiosis upon induction and to establish DNA methylation correctly for the limited number of igDMRs tested, their wider epigenetic fidelity has not been evaluated. We sought to optimise this oocyte culture system further to produce a larger quantity of GV stage oocytes for experiments. We evaluated mouse strain and age at commencement of culture (data not shown), settling on 7-day-old F_1_(C57BL6/Babr background) females. We included fatty acid-free bovine serum albumin (fafBSA), epidermal growth factor (EGF), and follicle stimulating hormone (FSH) in the culture medium, as these factors have been shown to improve oocyte development and maturation [25–27]. We also considered the effect of oxygen tension. Human oocytes in vivo are in contact with 3–5% oxygen [8], while in other mammals the oxygen concentration has been reported as being between 2 and 8% [28]. Most follicle cultures for mouse oocytes have been carried out under normoxia, with only a few studies using lower oxygen levels, such as 5% O_2_ [9]. In this study, we compared 20% O_2_ and 5% O_2_ (Fig. 1a).

To evaluate the performance of the cultures, and for subsequent molecular analyses, we size-selected oocytes and categorised them into four classes by their diameters: I, < 40 µm; II, 41–49 µm; III, 51–59; µm and IV, 60–70 µm (Fig. 1a). The progression of de novo DNA methylation has been described in in vivo oocytes of similar size classes [23], which serves as a reference. In the optimised normoxia cultures, oocytes achieved a maximum diameter after 14 days, whereas in 5% O_2_, size IV was attained in only 9 days and after these 9 days, the oocytes started to degenerate. Under both conditions, oocytes developed into GV-like oocytes similar to in vivo oocytes (Fig. 1b), but the maximum sizes attained differed. An in vivo fully grown GV measures 70–80 µm [29], whereas in 20% O_2_, only 7% of the oocytes reached a size of 65 µm and in 5% O_2_ 10% of oocytes achieved a size of 70 µm (Fig. 1c, *p* < 0.0001, 7 independent oocyte cultures for both conditions). The contingency graph describing the growth model showed that the number of oocytes under 20% O_2_ decreased slowly from class I–IV, while they grew, whereas in 5% O_2_ the oocytes maintained their number at a plateau, when the oocytes reached the size of 50–60 µm (from class II–III) and then slowly decreased in number to reach class IV (Fig. 1d , *n* = 7 independent oocyte cultures for both conditions).

During the final step of oogenesis, the oocyte nucleus is subject to large-scale chromatin modifications that correlate with transcriptional silencing. Oocytes that present uncondensed chromatin (NSN, non-surrounded nucleolus) are transcriptionally active, while oocytes with dense chromatin around the nucleolus are silent (SN, surrounded nucleolus); moreover, the transition to SN is required for developmental competence [30]. To define the pattern of chromatin organisation, the in vitro-grown oocytes were stained with DAPI and compared with in vivo GVs collected from 23- to 25-day-old females (3 independent oocyte cultures for both conditions, total of oocytes *n* = 68 for 5% O_2_ and *n* = 62 for 20% O_2_). Under either culture condition, IV-class oocytes comprised both NSN and SN GVs (Fig. 1d); however, the SN oocytes grown in 20% O_2_ had a bigger nucleolus than in 5% O_2_. In terms of the SN and NSN proportions, oocytes grown in 5% O_2_ condition were closer to those of in vivo GV oocytes (75% SN, compared with 71% in vivo GV, *n* = 31 oocytes from 23- to 25-day-old females; [30]), but only 40% of oocytes grown in 20% O_2_ were in the SN chromatin organisation.

### Characterisation of DNA methylation in in vitro-grown oocytes

**Fig. 2:**
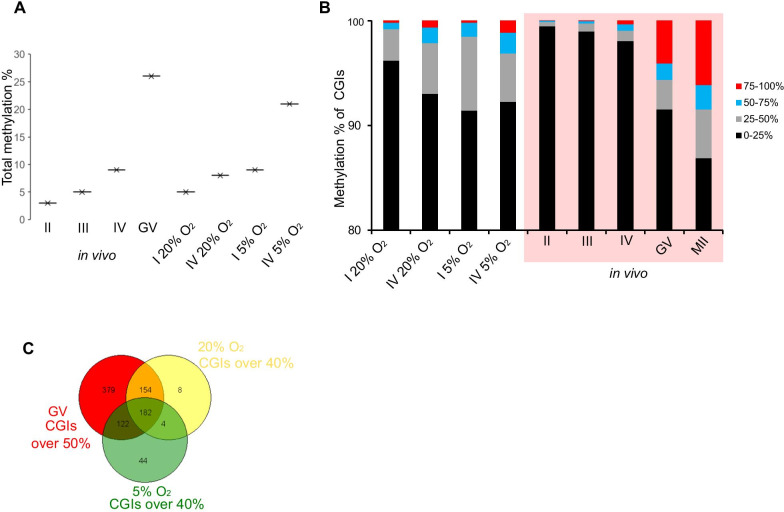
DNA methylation landscape in in vivo and in vitro oocytes. **a** Comparison of the total methylation level, **b** percentage of methylation at CGI between in vivo and in vitro oocytes (for II *n* = 16,473, III *n* = 16,151, IV *n* = 16,177, I 20% O_2_ *n* = 17,441, IV 20% O_2_ *n* = 17,170, I 5% O_2_ *n* = 17,256, IV 5% O_2_ *n* = 16,009). **c** Venn diagram showing the common CGIs that were affected in GV, 20% O_2_ and 5% O_2_ oocytes with different levels of methylation

DNA methylation acquisition in the oocyte correlates with increasing diameter during oogenesis [23]. Therefore, the DNA methylation level of the oocytes grown under the two culture conditions was compared with the methylation of in vivo size-selected oocytes. We collected oocytes representing the starting populations and the largest oocytes at the end of the cultures which, for practical purposes, corresponded to ≤ 50 µm and 55–65 µm for the 20% O_2_ cultures, and ≤ 40 µm and 60–70 µm in the 5% O_2_ cultures. Pools of ~ 250 for the class IV to ~ 500 oocytes for class I and II were collected in replicate and processed for reduced representation bisulphite sequencing (RBBS), which preferentially samples CG-rich regions in the genome, including CGIs [31]. RRBS data from the cultured oocytes were compared to published data from in vivo growing oocytes (I–IV [23] and GV [3]). When the replicates were merged depending on their size within the experimental groups, coverage was sufficient to evaluate between 56.4 and 60.1% of CGIs (≥ 5 CpG sites per CGI and ≥ 5 reads per CpG; CGI annotation based on [32]). Principal component analysis (PCA) indicated that replicates clustered together (Additional file 1: Fig. S1). Furthermore, PCA demonstrated a separation along PC1 of the in vivo groups corresponding to developmental stage. Both 20% O_2_ and 5% O_2_ samples also separated on PC1 according to oocyte size, indicative of a de novo methylation process similar to in vivo, but the 5% O_2_ samples also separated on PC2 from the other groups due to the effect of 5% O_2_. As previously reported [3, 4], total methylation of CpGs increased from 3.2% in group II in vivo oocytes to 10% in group IV and 26.3% at the fully grown GV stage (Fig. 2a). The in vitro-cultured oocytes also exhibited increases in methylation with size: from 5 to 8% for the 20% O_2_ cultures, and 9–21% for the 5% O_2_ cultures (Fig. 2a). These results suggest that growth in 5% O_2_ is more suitable for in vivo-like DNA methylation establishment than 20% O_2_.

**Fig. 3:**
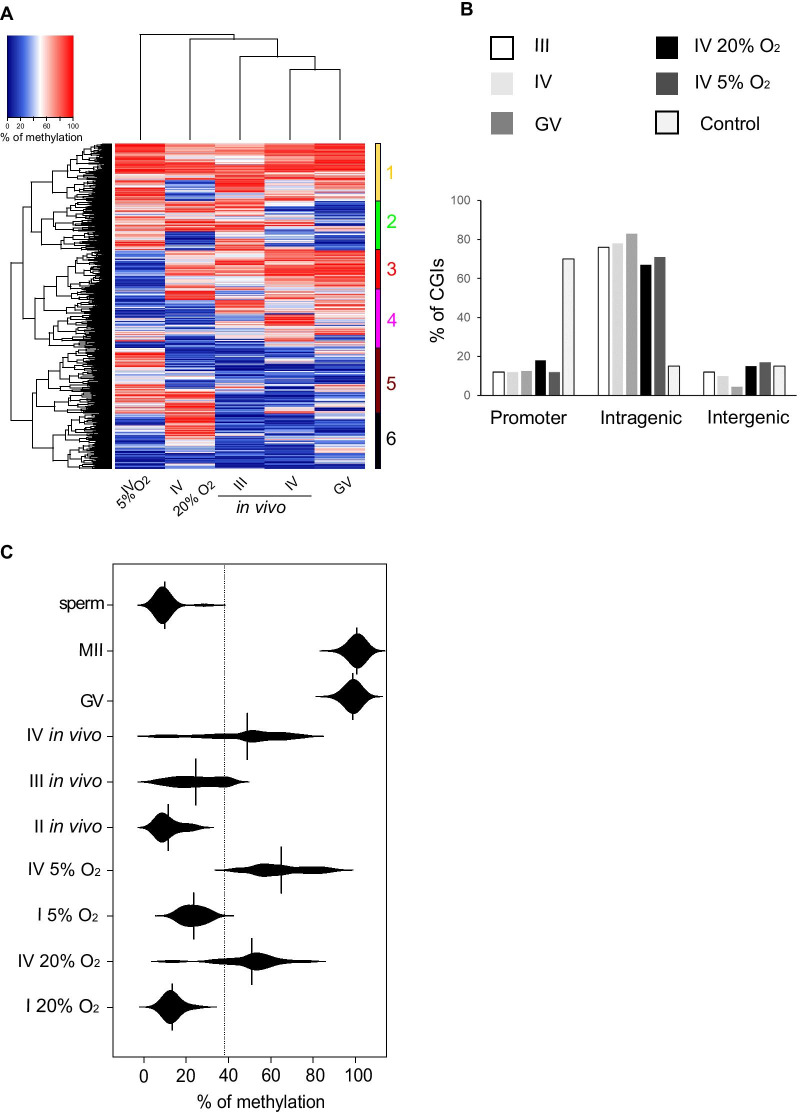
Differentially methylated CGIs. **a** Heatmap of over 40% of methylation in at least one sample (*n* = 1530) comparing IV 20% O_2_, 5% O_2_-grown oocytes with size-selected III, IV, and GV in vivo oocytes. **b** Distribution of CGIs over 40% of methylation in at least one sample (*n* = 1530) located overlapping promoter, intragenic, and intergenic regions. **c** Bean plot of the methylation level of the 23 igDMRs between in vivo and cultured oocytes

We next considered the fidelity of DNA methylation in the in vitro-grown oocytes. Similar to most cell types, the majority of CGIs are unmethylated in oocytes, but fully grown GV oocytes in vivo have a defined number of highly methylated CGI [3, 21, 22]: in the in vivo GV dataset we used as reference 4.1% of CGIs (*n* = 661) are hypermethylated (> 75%). In comparison, 1.2% (*n* = 169) of the CGIs informative in our RRBS datasets of the IV-sized oocytes from the 5% O_2_ cultures were classed as highly methylated, and just 0.06% (*n* = 9) of CGIs in oocytes grown in 20% O_2_ (Fig. 2b). On the other hand, 631 CGIs (4.6%) had methylation > 25% in the IV oocytes grown in 20% O_2_, and 1022 (7.45%) in 5% O_2_, which might indicate that the CGIs normally fully methylated in vivo are partially methylated in the in vitro-grown oocytes. To evaluate the CGIs subject to de novo methylation more closely, we selected those that had ≥ 40% methylation in at least one sample, which were 1530 of the 13,710 CGIs for which RRBS data were available in size III–IV and GV in vivo oocytes, and IV 20% O_2_ and IV 5% O_2_ oocytes (Additional file 1: Fig. S2). When the CGIs > 40% methylated in at least one sample including in vivo samples were clustered with the CGIs > 50% from GV stage oocytes, we observed that ~ 40% of the CGIs were common between GV and both in vitro conditions (Fig. 2c). With the exception of a single CGI found in the sample 5% O_2_, all CGIs ≥ 40% methylated in in vitro-grown oocytes were found in > 25% methylated in GVs (Additional file 1: Fig. S3A). To confirm that CGIs > 40% methylated in at least one sample were a correct cut-off from the total CGIs for further analysis, we correlated CGIs > 40% methylated in at least one sample with CGIs of GV, IV in vivo, III in vivo, IV 5% O_2_ and 20% O_2_. The correlation coefficient between GV and IV in vivo was lower than the correlation coefficient between IV in vivo and III in vivo, confirming the results observed by Gahurova et al*.* [23]. The correlation coefficient between IV in vivo and 20% O_2_ was better than IV in vivo and 5% O_2_ confirming the PCA plot (Additional file 1: Fig. S3B). From unsupervised hierarchical clustering analysis of the 1530 CGIs, six major clusters were apparent (Fig. 3a). Cluster 1 generally comprised CGIs methylated in all samples; cluster 2 had methylated CGIs in in vitro conditions and Class III in vivo, but lacking the methylation in class IV and GV in vivo samples; clusters 3 and 4 were dominated by CGIs showing progressive methylation in vivo samples but lacking methylation in the 5% grown oocytes; clusters 5 contained CGIs methylated predominantly in the in vitro-grown oocytes; and cluster 6 grouped all the intermediate methylated CGIs in all samples except GV in vivo samples (Fig. 3b). For all of the samples, CGIs > 40% methylated were predominantly intragenic in location, compared with unmethylated CGIs (Fig. 3B), consistent with the dependence of CGI methylation on transcription events in oocytes [3, 4].

Amongst the genomic elements for which de novo DNA methylation in oocytes does have a critical role are the igDMRs of imprinted genes. Oocytes grown either in 5% O_2_ or 20% O_2_ demonstrated a gain of methylation at igDMRs comparable to the corresponding sized in vivo oocytes (Fig. 3c), with the oocytes grown in 5% O_2_ showing a higher mean methylation level (58.1% compared with 42.9%), suggesting greater progression towards the complete methylation characteristic of fully grown GV oocytes. Together, these results confirm that DNA methylation starts to be established in the in vitro cultures, with critical elements gaining methylation appropriately, but there are also subsets of CGIs displaying anomalous methylation.

### Transcriptome analysis of in vitro-grown oocytes

To investigate gene expression patterns in the oocytes grown in vitro, we generated deep, strand-specific libraries in duplicate from the same size populations of in vitro-grown oocytes as used in the methylation analysis. PCA of these RNA-seq libraries, in comparison with in vivo reference datasets [23], indicated that the in vitro-grown oocytes from either O_2_ concentration separated from in vivo growing oocytes and were closer to GVs on PC1, but were separated from all in vivo groups on PC2 (Additional file 1: Fig. S4).

**Fig. 4:**
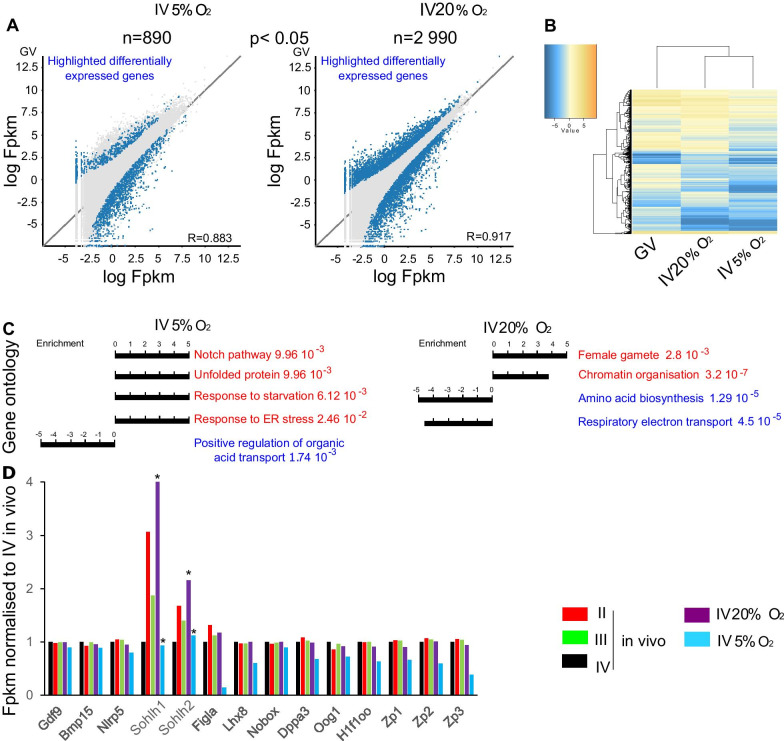
Gene expression profiles of grown IV oocytes. **a** Scatter plot and correlation coefficient of log Fpkm values of 890 transcripts in GV and 5% O_2_, and of 2990 transcripts in GV and 20% O_2_. **b** Heatmap of the transcripts from the IV 5%O_2_, IV 20% O_2_, and GV. **c** Gene ontology of the differentially expressed genes between GV and 5% O_2_ and GV and 20% O_2_. **d** Gene expression of selected oocyte genes in in vivo, IV 20% O_2_ and IV 5%O_2_. *p > 0.05 Student’s t test comparing GV and the cultured oocytes

**Fig. 5:**
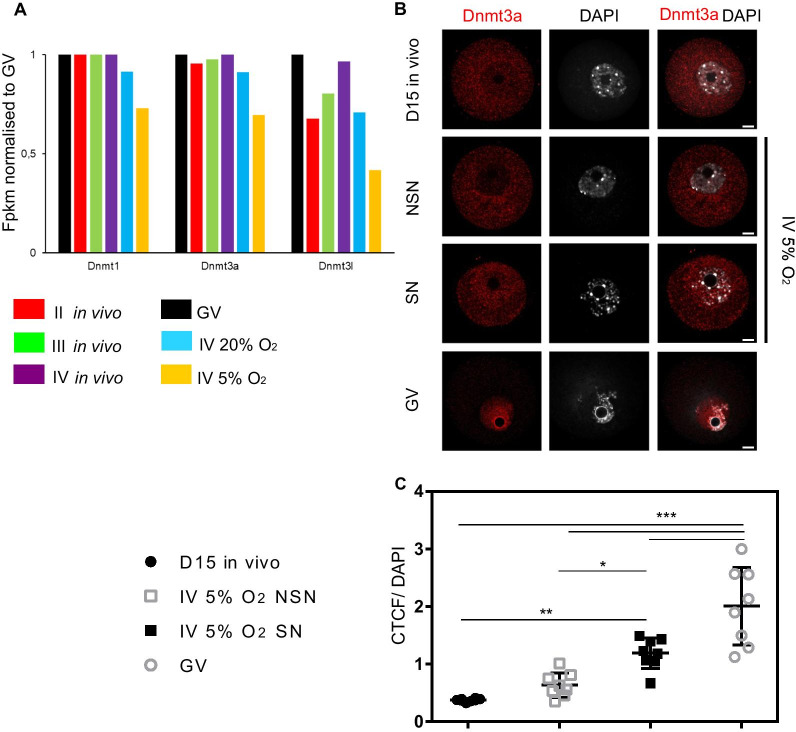
DNA methyl transferase expression in grown oocytes. **a** *Dnmt1*, *Dnmt3a*, and *Dnmt3l* expression compared to GV. *p > 0.05 Student’s t test GV compared to cultured oocytes. **b** Immunostaining of Dnmt3a in different size oocytes.  **c** Quantification of immunofluorescence intensity as seen in (**b**). Corrected total cell fluorescence (CTCF) was calculated for each signal then normalised to DAPI CTCF. Bars represent mean values, and error bars show SEM (2-way ANOVA test; *p < 0.05; **p < 0.01, ***p < 0.001)

To explore the gene expression differences between in vitro-grown and in vivo oocytes, we used DESeq to compare the in vitro RNA-seq data with in vivo GVs. The global gene expression correlations between the IV 5% O_2_ oocytes and GVs, and between the IV 20% O_2_ oocytes and GVs, were *r* = 0.883 and *r* = 0.917, respectively (Fig. 4a, b); furthermore, there were 890 differentially expressed genes between IV 5% O_2_ and in vivo GVs, and 2990 between IV 20% O_2_ and GVs (Fig. 4a, b, *p* < 0.05). Gene Ontology (GO) analysis using Panther revealed that the differentially expressed genes were most enriched in biological processes related to the unfolded protein response, response to starvation, and endoplasmic reticulum stress for IV 5% O_2_ oocytes, and terms related to female gamete and chromatin organisation for IV 20% O_2_ oocytes (Fig. 4c). We also interrogated the expression of oocyte-specific genes important for follicle development, reproduction, and early development [33]. In the 20% O_2_-grown oocytes, there was a greater abundance of transcripts for key oocyte transcription factors *Spermatogenesis And Oogenesis Specific Basic Helix-Loop-Helix 1 and 2* (*Sohlh1* and *Sohlh2*), whereas 5% O_2_-grown oocytes had reduced abundance of *Folliculogenesis-Specific BHLH Transcription Factor* (*Figla*) and *LIM Homeobox 8* (*Lhx8*) transcripts where both transcription factors co-express in oocyte for its maturation, as well as for the oocyte markers *Zona pellucida* genes, *Zp1*, *Zp2,* and *Zp3* (Fig. 4d). We also evaluated expression of *Dnmt3A*, *Dnmt3Ll* and *Dnmt1* which are key enzymes for regulating the establishment of methylation on DNA in the RNA-seq datasets. Transcripts for all three genes had reduced abundance in the 5% O_2_ and 20% O_2_-grown oocytes, with a greater reduction in the 5% O_2_-grown oocytes (Fig. 5a, *p* < 0.05 for *Dnmt1* gene, isoform *Dnmt1o* oocyte specific). For Dnmt3a, the principal catalytically active de novo methyltransferase in mouse oocytes, we also examined protein abundance and localisation by immunofluorescence, comparing IV 5%O_2_ oocytes (SN and NSN) with in vivo post-natal D15 growing oocytes and GV oocytes. Dnmt3a protein was observed both in the cytoplasm and nucleus in the NSN oocytes at D15, whereas in SN GV oocytes it was concentrated on chromatin, accompanied by an increase in total protein abundance (Fig. 5b, c). In the NSN oocytes from the 5% O_2_ cultures, Dnmt3a was predominantly cytoplasmic and retained some cytoplasmic localisation even in the SN-like oocytes (Fig. 5b, C).

Finally, because of the role of transcription events in determining DNA methylation in growing oocytes [4, 21], we integrated results from the RRBS and RNA-seq from the cultured oocytes. After stratifying intragenic CGIs for level of DNA methylation attained, we detected a positive correlation between gene expression level and methylation, particularly for the oocytes grown in 20% O_2_ (Additional file 1: Fig. S5), suggesting that CGIs more advanced in de novo methylation are located within more highly expressed genes. Therefore, although the in vitro-grown oocytes do not completely recapitulate the DNA methylation profile of in vivo counterparts, the de novo methylation mechanism is likely to follow the same mechanistic principles.

### Testing elements of the de novo methylation mechanism in the in vitro culture system

To investigate the utility of the oocyte culture system and to investigate mechanisms of DNA methylation establishment, we conducted an experiment using two drugs: tranylcypromine hydrochloride [34] and lithium chloride (LiCl) [35]. Tranylcypromine hydrochloride is a non-selective monoamine oxidase inhibitor (MAO) and can be used to inhibit an amine oxidase reaction essential for the activity of the histone lysine demethylases of the KDM1 family [36, 37], which have activity towards H3K4me2. Both Kdm1a and Kdm1b have roles in promoting normal de novo DNA methylation in growing oocytes [38, 39], in part by demethylation of H3K4me2 which is antagonistic to Dnmt3a binding and activity [40]. LiCl was chosen because it blocks activity of the oocyte-specific transcription factor Foxo3a by inhibiting its phosphorylation and translocation to the nucleus [35]. In view of the greater apparent fidelity of methylation in oocytes grown in 20% O_2_, we selected to culture the oocytes under normoxia. To evaluate the efficacy of MAO treatment, we performed immunofluorescence for H3K4me2 and H3K4me3 on oocytes after 14 days of culture, observing that H3K4me2 and H3K4me3 staining was reduced (Additional file 1: Fig. S6A, *n* = 10 oocytes for control and treated samples). For the LiCl-treated oocytes, the nuclear staining of Phospho-FOXO3A, that is seen in the untreated oocytes, is much diminished (Additional file 1: Fig. S6B, *n* = 10 oocytes for control and treated samples).Along with the staining of the cultured oocytes, GV oocytes (*n* = 8 for each condition MAO, LiCl, and control) were stained to observe the correct localisation of proteins of interest to assure that the 20% O_2_ had no effect on the cultured oocytes (Additional file 1: Fig. S6C).

To quantify the effect of MAO and LiCl treatments on DNA methylation, we collected oocytes after 14 days of culture, grown under 20% O_2_, in the absence or presence of the inhibitors in triplicate pools of between 70 and 110 oocytes, and processed the oocytes for whole-genome methylation analysis by low-cell post-bisulphite adaptor-tagging (PBAT) [38, 41]. After alignment and removal of reads from duplicates between 16,798,787 and 45,690,308 uniquely mapped reads were obtained per library. (One LiCl-treated sample was discarded because of low coverage, Additional file 2: Table S1.) For the clustering analysis, we included in this analysis, the Class IV in vivo oocyte from Gahurova et al. [23] to identify how the samples will group, as the class IV oocytes is our aimed size and present the maximum level of methylation. The PBAT libraries clustered according to the treatment (Additional file 1: Fig. S7). For most subsequent analysis, replicates were pooled.

**Fig. 6:**
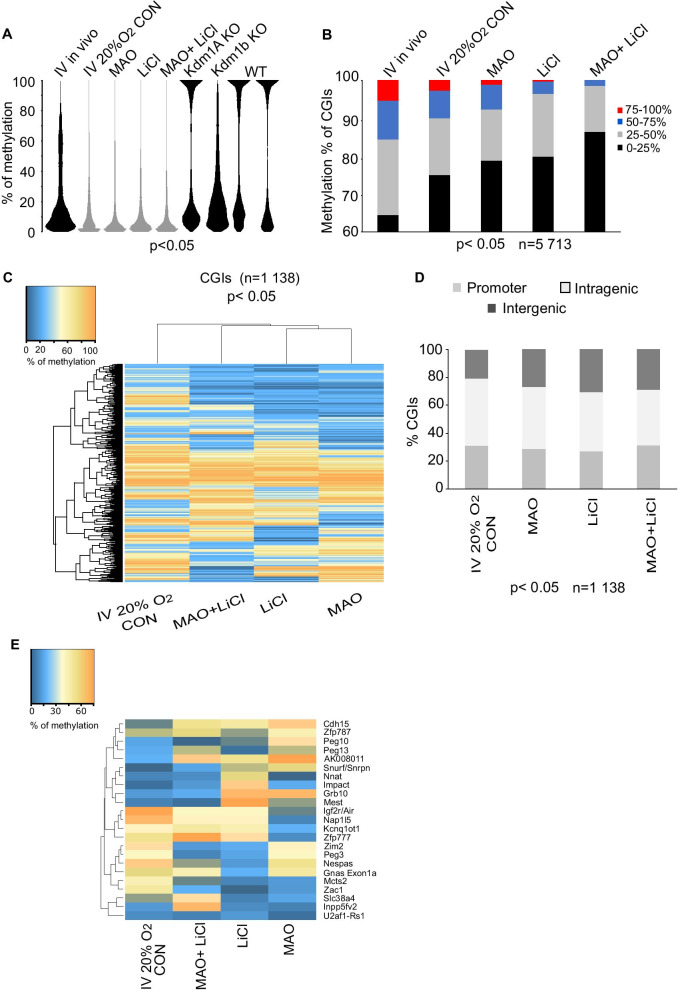
CGI methylation under four different conditions using oocyte in vitro model. **a** Bean plots representing total methylation in IV in vivo, IV 20% O_2_, MAO, LiCl, MAO + LiCl-grown oocytes and *Kdm1a*, *Kdm1b* knock-out and WT M2 oocytes, (*χ*^2^ test, *p* < 0.05). **b** Percentage of methylation at CGIs between in vivo and cultured oocytes (for IV in vivo *n* = 5707, IV 20% O2 CON *n* = 5713, MAO *n* = 5713, LiCl *n* = 5713, MAO + LiCl *n* = 5713, *χ*^2^ test, *p* < 0.05), **c** Heat map of the CGIs over 40% of methylation in at least one sample between the conditions, (*n* = 1138; *χ*^2^ test, *p* < 0.05). **d** Distribution of CGIs over 40% of methylation in at least one sample (*n* = 1138) located overlapping promoter, intragenic, and intergenic regions (*χ*^2^ test, *p* < 0.05). **e** Heatmap representing the quantitation of the methylation difference between the IV 20% O_2_ CON compared to each cultured in vitro oocytes of the 23 igDMRs (*χ*^2^ test, *p* < 0.05)

First, we assessed the effects of the inhibitors on overall DNA methylation in the oocytes and observed a decrease in CpG methylation in the three different conditions from 39.7% (MAO), 28.8% (LiCl) to 19.6% (MAO + LiCl) compared to 46.6% in in vivo IV oocytes, and 38% in 20% O_2_ condition (Fig. 6A). As one of the treatments affects the activity of Kdm1 enzymes, we included the PBAT methylation datasets of *Kdm1a* and *Kdm1b* M2 knock-out oocytes from Stewart et al*.* [38] in the analysis. Treatments of MAO or MAO + LiCl showed a reduced CpG methylation with a pattern close to Kdm1b (data not shown). However, here we compared oocytes from different sizes (Class IV in vitro oocytes 20% O_2_ with M2 ovulated oocytes) and could bias the results. Focussing on CGIs that normally become methylated in oocytes, we then access the total CGIs methylation which showed a reduction in highly methylated CGIs from 1.3% (*n* = 76) in 20% O_2_ CON to nearly 0% in MAO (*n* = 32), LiCl (*n* = 8), and MAO + LiCl (*n* = 2). The number of methylated CGI < 25% was increasing in all treated samples and was of 10% between 20% O_2_ CON and MAO + LiCl (Fig. 6B). Then, we used the same strategy as for analysing the RBBS data, and a cut-off of > 40% methylation was used on the PBAT data set. This resulted in 1138 CGIs which clustered within 3 groups and were mostly hypomethylated in the cultured oocytes under the inhibitory drugs (Fig. 6c). They failed to properly methylate, and the proportion of CGIs localised on the promoter and intergenic regions increased compared to oocyte 20% O_2_ CON. Most of the methylation is observed on the intragenic regions in GV oocytes [4], and the cultured oocytes presented a significant decreased in CGIs at the intragenic regions from 48% in 20% O_2_ CON to 40% in cultured oocytes with Mao + LiCl (Fig. 6d). We also specifically evaluated effects on igDMRs, as it has previously been described that genetic ablation of *Kdm1b* in oocytes [32] impairs de novo methylation of igDMRs. The heatmap of methylation difference of the igDMRs by comparing 20% O_2_ CON with each drug presented a various aberrant igDMRs methylation for all the cultured oocytes (Fig. 6e). For example, Igf2r was highly methylated (70.1%) in IV 20% O2 CON and the cultured oocytes under treatment reduced their methylation (MAO 56.5%, LiCl 38.2%, and MAO + LiCl 33.9%, respectively, Additional file 1: Fig. S7). These results suggest that the igDMRs did not gain a correct level of DNA methylation but instead obtained various levels of intermediate methylation.

**Fig. 7:**
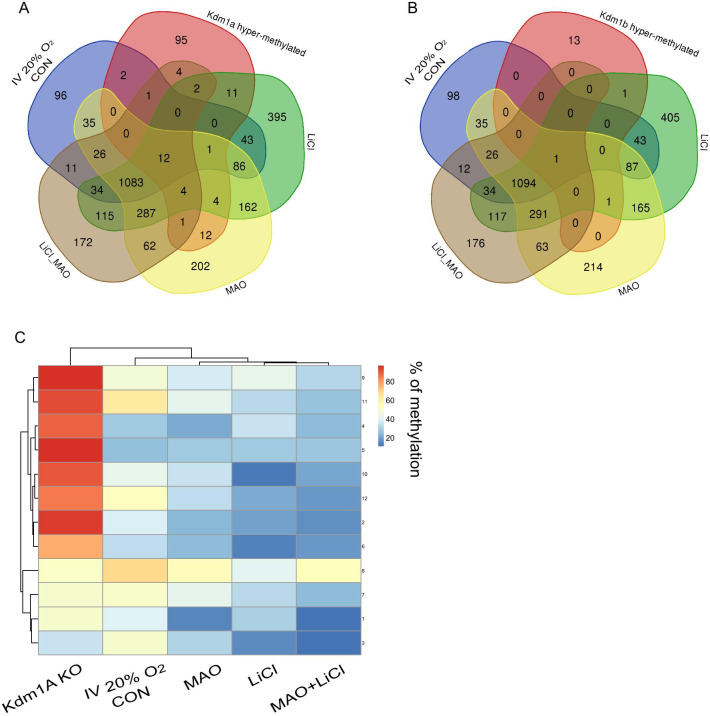
Identification of common CGIs between *Kdm1s* knock-out mouse M2 oocytes and in vitro cultured oocytes under treatments inhibiting methylation and transcription. **a** Venn diagrams showing the common and non-common CGIs between the hypermethylated domains defined in *Kdm1a* oocytes and the CGIs methylated > 25% in normoxia or 20% O_2_ CON and > 10% in MAO, LiCl, and MAO + LiCl. **b** Venn diagrams showing the common and non-common CGIs between the hypermethylated domains defined in *Kdm1b* oocytes and the CGIs methylated > 25% in normoxia or 20% O_2_ CON and > 10% in MAO, LiCl, and MAO + LiCl. **c** Heatmap representing the methylation level of 12 common CGIs between *Kdm1a* M2 oocytes and the CGIs methylated > 25% in normoxia or 20% O_2_ CON and > 10% in MAO, LiCl, and MAO + LiCl

Finally, we sought to assess the extent to which the treatments in vitro mimicked the ablation of *Kdm1a* and *Kdm1b *in vivo [38]. We took advantage of the published PBAT data from M2 oocytes of *Kdm1a* and *Kdm1b* knock-out mice [38]. We compared the defined hypermethylated domains (CGI over 75% methylation) of M2 oocytes knock-out mice, parameters defined in Stewart et al*.* [38], with our data at IV 20% O_2_ CON oocytes > 25% methylation and the 3 other in vitro conditions (MAO, LiCl, and MAO + LiCl) over 10% of methylation. We have chosen a methylation threshold of ≥ 25% methylation for 20% O_2_ CON (*n* = 1433) as most of the CGIs had an intermediate methylation. For the other in vitro conditions MAO, LiCl, and MAO + LiCl, the methylation threshold was chosen ≥ 10% methylation to gain the maximum number of CGIs in the different culture conditions (MAO *n* = 1983, LiCl *n* = 2243, and MAO + LiCl *n* = 1817). We have generated Venn diagrams to identify the overlapping CGIs between the effect of the in vitro conditions and the in vivo knock-out mice dataset. While comparing the CGIs in hypermethylated domains with in vivo CGIs of *Kdm1a* knock-out mouse hypermethylated domains, only 12 CGIs were common with all the in vitro conditions. When same comparison was carried out with the in vivo CGIs of *Kdm1b* knock-out mouse hypermethylated domains, only one CGI was common (Fig. 7a–c). In the case of the comparison with hypermethylated domains *Kdm1a* knock-out mouse, the 12 common CGIs are located mostly on intragenic regions (75%) and the remaining 25% are located on the promoter (Additional file 3: Table S2). However, in the case of non-common CGIs, we could observe an increase of CGIs located on intergenic regions for all conditions compared to *Kdm1a* and *Kdm1b* M2 knock-out oocytes (Additional file 3: Table S2). Interestingly, the CGIs in hypermethylated domains of 20% O_2_ CON compared with hypermethylated domains of *Kdm1a* knockout oocytes (*n* = 96) and the CGIs from 20% O_2_ CON compared with hypermethylated domains of *Kdm1b* knock-out oocytes (*n* = 98) were all common (Additional file 3: Table S2). These results suggest that the drugs against Kdm1 enzyme activity slowed down the gain of methylation on CGIs in grown oocytes.

## Discussion

Reproductive biology and regenerative medicine fields have made great breakthroughs to understand how to preserve fertility. Reconstitution of gametogenesis in vitro is crucial for improving and preserving fertility. To be able to reconstitute mature gametes, different alternatives have been explored to generate live offspring based on producing gametes from pluripotent stem cells [42] or from embryonic mouse germ cells [43]. However, it is essential to prove the safety of such procedures before they become applied in humans by analysing and understanding the effect of the cultures on the epigenetic profile of the gametes. Towards this goal, we have analysed the effect of oxygen concentration (5% O_2_ vs. 20% O_2_) on de novo methylation establishment and transcription in in vitro-grown GV-like oocytes from an in vitro oocyte culture starting from immature oocytes.

Most of the follicle culture techniques are using super-ovulated oocytes (M2) [44] when they are carried on animal and human oocytes. In these culture methods, all follicles are grown with the cumulus cells around the oocytes, and the connections between the oocyte and cumulus cells are intact. In our model, few granulosa cells were attached to the immature oocytes due to the ovaries digestion step and the free theca cells resulting from the ovarian digestion formed colonies at the bottom to the Petri dish as described by Honda et al*.* [24]. By adding identified factors (fafBSA, EGF, and FSH) in the culture medium, known to improve oocyte development and maturation [25–27], class IV in vitro oocytes were collected. A small amount of class IV grown oocytes under 20% O_2_ (7%) and 5% O_2_ (10%) reached the size of GV in vivo oocytes which was the aimed size for our study. The oocytes that reached class IV under both oxygen conditions demonstrate that they complete the transition from the NSN to SN chromatin configuration to be mature oocytes [24].

Methylation in in vivo mouse oocytes was first identified to be crucial in intragenic CGI using RRBS [3] and then more recently using whole genome bisulphite sequencing via PBAT method on low amount of sample amount [21, 22, 38]. Veselovska has been demonstrating that methylation happens at the same time as transcription is occurring [4] and methylation of the last CGIs depends on the chromatin state [23]. However, the detail of the genomic distribution of methylation in in vitro mouse oocytes has not yet been clearly analysed. Here, we provide DNA methylation maps of mouse grown oocytes from immature to GV stage obtained after 9–14 days in culture under 5% O_2_ and 20% O_2_ condition. We observed that DNA methylation on CGIs was increased in all conditions as we could identify the methylation wave happening in the in vitro conditions. However, the hypermethylated CGIs regions were altered in the class IV in vitro oocytes in both conditions due to the oxygen concentration, 5% and 20% O_2_. We can hypothesise that a longer period of culture (up to 14 days) in relation to the oxygen concentration might affect the methylation status of the cytosine base and a demethylation wave might be triggered towards hydroxymethylation, resulting in hydroxymethyl cytosine and even further to the formyl cytosine. Therefore, such study needs to be carried out to portray the importance of reduced oxygen tension in the establishment of demethylation wave in in vitro-grown oocytes.

Studies exploring the influence of the duration of the culture on M2 oocytes in close contact with cumulus cells demonstrated that the blastocyst formation rate, meiosis resumption, and expression of key oocyte genes are increased [45], but their effects on imprinted genes are currently not fully known. In our study, the two different oxygen conditions (20% vs. 5% O_2_) did not affect the temporal acquisition of correct imprinted DNA methylation patterns in the denuded oocytes, as the methylation of the imprinted genes was closer to the class IV in vivo oocytes as the igDMR methylation was restored in in vitro 5% and 20% O_2_ oocytes. This result is in line with previous studies using in vitro culture model or super-ovulation method demonstrating that imprinted DNA methylation acquisition in oocytes is a robust mechanism and is not influenced by culture conditions [19, 44]. Imprinted genes are regulated by the methylation status of the imprinting control region (ICR), and when the methylation of the ICR failed due to the loss of transcription, it results in aberrant methylation of the igDMRs [46]. This is what we observe when we manipulated methylation and transcription processes at the same time by inhibiting Kdm1 function and phosphorylation of Foxo3 transcription factor (MAO + LiCl treatment). The igDMR methylation was completely altered underlining that the establishment of methylation on imprinted genes might be dependent on the transcription.

To further investigate the facets of methylation and transcription, we generated and compared the methylomes and transcriptomes of in vitro-grown oocytes to in vivo size selected and class IV in vivo oocytes. While DNA methylation establishment at igDMRs in the oocyte remained unaltered, the overall distribution of CGI methylation occurred in a similar pattern that in in vivo oocytes during the oocyte growth; however, we observed that the methylation in intergenic region increased. Recent study shows that one-sixth of all de novo DNA methylation is linked to transcription initiated at long-terminal repeats (LTRs) [47] and most of the LTRs are localised in intergenic regions. We can hypothesise that if the LTR-initiated transcripts are activated at a wrong time, this will impact the methylome in oocytes leading to aberrant methylation. Transcription analysis revealed that in 20% O_2_ culture condition, transcripts were enriched in female gamete genes and chromatin organisation, whereas the 5% O_2_ condition increased expression of stress response genes. Such results suggest that the lower oxygen concentration towards hypoxia might lead to an exhaustion of metabolites in the oocytes resulting in mechanical stress and/or increased of reactive oxygen species, and normoxia condition to a better availability of the 3D chromatin conformation. However, the Dnmts transcripts (Dnmt1, Dnmt3A, Dnmt3L) were reduced in both oxygen conditions 20% and 5% O_2_. De novo DNA methylation is established by the Dnmt3A/L complex, and such enzymes are controlled by their availability at the subcellular localisation and by their interactions with the chromatin. They regulate the methylation at the hypomethylated and hypermethylated domains in oocytes [48]. We observed in our in vitro oocyte model that the localisation of Dnmt3A protein was altered in 5% O_2_-grown oocytes. Dnmt3A was observed in the cytoplasm in class IV in vitro oocytes, whereas it should have been localised onto the chromatin as in in vivo class IV oocytes. Such mis-localisation will lead to reduce catalytic activity of the enzyme and might explain the lower methylation level observed at the hypermethylated domain in grown oocytes.

The clinical implication of low oxygen concentration in human embryos in assisted reproductive technology has been analysed mostly at the level of embryo quality and morphology, pregnancy rate, live birth/ongoing pregnancy [49–51]. These studies show that in human low oxygen concentration might have some beneficial effect in the clinical outcomes of in vitro culture such as better pregnancy rate. So far, no studies have been conducted on analysing epigenetic marks such as DNA methylation in cultured human oocytes incubated for few hours in 5% O_2_ or in cultured blastocysts after 4–6 days incubation at 5% O_2,_ due to the difficulty to obtain the samples. Metadata analysis on human cohorts has hypothesis that changes in DNA methylation at specific genes during early embryo development might result in metabolic diseases in adult life such as obesity and diabetes [52]. To be able to answer such hypothesis, more studies need to be carried out to help us in understanding how DNA methylation is fully established in cultured oocytes and if any culture conditions during fertility treatments might affect the epigenome of the embryo. Therefore, whether the culture of human oocytes and embryos in low concentrations of oxygen can actually improve the clinical outcomes of ART is a question that still requires more data.

## Conclusions

In conclusion, the comparison of our in vitro oocyte models from immature towards class IV oocytes demonstrates that genomic DNA methylation is altered during the oocyte growth under low concentration of oxygen. Importantly, we show that CGI methylation of igDMRs is fully conserved, whereas the CGI methylation on gene bodies is disturbed due to alteration in culture conditions, here the oxygen concentration. Therefore, from our results further study on embryos produced from immature grown oocytes under different conditions is needed to clarify whether oocyte-derived DNA methylation at igDMRs and DMRs is fully conserved and if the future embryos develop properly.

## Material and methods

### In vitro oocyte culture

The oocyte culture established by Honda et al. [24] based on stem cell factor and ES medium was optimised. In brief, the ovaries, aged of 7 days old, from C57 Black6 background breed with SV129 background mice were collected and digested with trypsin (Sigma) and collagenase I (Sigma) at 37 °C for 15 min. The trypsin-collagenase action was stopped with foetal bovine serum (Sigma) and centrifuged for 5 min at 1600 rpm. The pellet was suspended into warm medium and plated onto a gelatine coated plate. The oocyte culture was performed under two different conditions: 20% O_2_ and 5%O_2_ (Fig. 1a). The culture medium was changed every 24 h for the 5% O_2_ as the colour of the medium changed rapidly to maintain the integrity of the medium composition, and every 48 h for the 20% O_2_ condition. The grown oocytes were collected depending on their size and the culture condition and were kept in—80 °C until further process. To reach the appropriate growth size, the oocytes were grown for 9 days under 5% O_2_ and for 14 days under 20% O_2_. The size of the oocytes has been grouped into classes: < 40 µm class I, 41–49 µm class II, 50–59 µm class III, and 60–70 µm class IV (Fig. 1a). For 5% O_2_, the size of the oocytes was collected at < 40 µm considered as class I and 60–70 µm class IV, and for 20% O_2_, the selected size of the oocyte was < 49 µm class II and 55–65 µm considered to be class IV as the diameter of these oocytes reached 65 µm.

Oocytes were cultured with 100 µM tranylcypromine (Sigma), a working concentration which has been shown to inhibit KDM1, and cause epigenetic changes at the promoters of specific genes [53] and with 15 mM/ml LiCl (Sigma) [35] under 20% O_2_ condition. The control oocytes (CON) were cultured without any drugs under 20% O_2_ condition. The oocyte cultures were stopped when the oocytes reached the size of 55–65 µm. The cultured oocytes were frozen at − 80 °C until further experiments.

### Preparation of bisulphite sequencing libraries

For RBBS, the oocytes were collected in pools of between 250 and 500 oocytes depending on the size of the oocyte and were carried in replicate for each condition 20% O_2_ and 5% O_2_ at two different oocyte size (class I and IV). Libraries for RRBS were prepared as described previously by Smallwood et al*.* [3]. Genomic DNA from approximately 300 cultured oocytes was extracted using QIAamp DNA Micro Kit (Qiagen) and was spiked with 10 fg of unmethylated lambda phage DNA (Promega) followed by *MspI* digestion (Fermentas), and the libraries were generated according to Smallwood et al*.* [3]. Libraries runs, in duplicates, were single-ended, and 100 base pairs (bps) in length were sequenced on Illumina HiSeq 2500 platform.

Post Bisulfite Amplification Treatment (PABT) libraries were generated as described previously by Peat et al*.* [41] for whole genome sequencing. 70–100 cultured oocytes under different treatments (Tranylcypromine, LiCl, Tranycyltramine + LiCl, and control also called 20% O_2_ CON) with a size of 55–65 μm were used to generate the libraries in triplicate. Libraries runs were single-ended, and 100 bps in length were sequenced on Illumina HiSeq 2500 platform.

### Preparation of RNA sequencing libraries

Total RNA from 280 cultured oocytes for each condition 20% O_2_ and 5% O_2_ at two different oocyte size (class IV) was extracted using trizol method (Sigma) according to the manufacturer’s instructions, and the total RNA was purified using the RNA clean and Concentrator-5 kit (Zymo) followed by depletion of ribosomal RNA with Ribo-Zero magnetic kit (Illumina) to remove the ribosomal RNA. The total RNA-seq libraries were constructed according to Veselovska et al*.* [4]. Libraries runs, in duplicate, were sequenced as paired-ended, 100 bps in length on Illumina HiSeq 2500 platform.

### Library mapping

Sequence alignment mapped onto the mouse genome (mm10) and methylation calls were performed using Bismark [54]. CpGs with read depth lower than 5 were discarded. We estimated bisulphite conversion rates using reads that uniquely aligned to the lambda phage genome (conversion rate over 94%). Raw reads from RNA-seq were aligned against mouse genome (mm10) and mapped using TopHat.

### Data analysis

DNA methylation and RNA analysis were done using the SeqMonk software package (v.1.41; Babraham Institute). To score CGI methylation, a list of 23,021 CGIs were obtained from a previous report [32]. The definition of methylated and unmethylated CGIs was according to Smallwood et al*.* [3]. A list of 23 DMRs was obtained from Proudhon et al*.* [55]. Methylation data were compared to RRBS data from size-selected in vivo oocytes (40–45 µm, 50–55 µm, 60–65 µm) [23] and the data from the GV and M2 from Smallwood et al*.* [3]. CGIs were used for methylation analyses if the minimum number of reads to count a position/minimum number of positions to count a probe was 5/5 for CGIs. CGI was annotated as (1) promoter if they overlapped a TSS defined as − 1 Kb + 500 bp, (2) intragenic if they overlapped a gene, and 3) intergenic if they did not overlap a promoter or intragenic regions.

PBAT methylation data were compared to data from GV oocytes, *Kdm1a-, Kdm1b-* knock-out, and wild-type M2 mouse oocytes [21, 22, 38]. The methylation level reported for a sample was the average methylation across samples from the generated biological triplicate samples. The minimum number of reads to count a position/minimum number of positions to count a probe was 5/3 for CGIs, followed by Chi-square test of *p* < 0.05 and probes which did not cover the requirements were discarded. The comparison with the PBAT dataset of *Kdm1a* and *Kdm1b* knock-out M2 oocytes, the strategy developed by Stewart et al*.* [4], was applied to the dataset to generate hypomethylated and hypermethylated domains.

For RNA-seq analysis, we used the annotation [4] that is transcripts oocyte-specific annotation. We then uploaded into SeqMonk as an oocyte-specific annotation track. Reads over transcripts were merged across exons correcting for feature length from the annotation track, quantitated using RNA-seq quantitation pipeline, and log2-transformed. Differentially expressed genes were called using DESeq with significance threshold of *p* < 0.05. Panther program was used for the gene ontology using Benjamini-corrected *p* < 0.05 significance cut-off. For gene-level analysis, the expression level of unique transcripts was ranked by expression levels (calculated as FPKM values) in each library. RNA-seq data from GV oocytes were from Veselovska et al*.* [4].

### Whole-mount immunofluorescence

Cultured oocytes under 5% O_2_, 20% O_2_, and in vivo D15 and GV oocytes from C57 Black6 background breed with SV129 background mice were collected, fixed in 4% paraformaldehyde for 15 min, and washed three times in PBS, and the immunofluorescence protocol was according to Santos et al. [56]. The oocytes were incubated with Dnmt3a (Abcam), H3K4me2/3 (Abcam), phospho-Foxo3a (Molecular probe), Fig alpha (Santa Cruz), or Alexa Fluor antibodies (Molecular probes) and were counterstained with DAPI (Sigma). The pictures were taken with Leica LSM confocal microscope. To accurately compare fluorescent intensity between oocytes, the laser power was set based upon the oocyte with the strongest signal and the same setting was used to evaluate all oocytes. Signal quantification was performed using NIH Image J software; corrected total cell fluorescence (CTCF) was calculated as CTCF = Integrated density—(Area of selected region X Mean fluorescence of background readings) [57]. CTCF was calculated for Dnmt3a and DAPI signals; Dnmt3a was normalised to the DAPI signal for each oocyte nucleus. Greater than 10 oocytes pooled for each condition were examined in 3 separate experiments.

## Supplementary Information

**Additional file 1**. Supplementary materials (Figures S1–S8).

**Additional file 2: Table S1.** Sequencing output for PBAT libraries.

**Additional file 3: Table S2.** CGIs overlapping promoter, intragenic and intergenic regions of methylated non-common CGIs after the comparison with the hypermethylated domains defined in *Kdm1a* oocytes and the CGIs methylated >25% in normoxia or 20% O2 CON, and >10% in MAO, LiCl and MAO+LiCl. *PA* promoter, *Intra* intragenic and *Inter* intergenic.

## Data Availability

Mapped sequence data from Bismark software have been deposited in the Gene Expression Omnibus database (GEO) under accession code GSE164864.
