# Supplementary material for: Oxygen concentration affects de novo DNA methylation and transcription in in vitro cultured oocytes
Source: Clin Epigenetics. 2021 Jun 28;13:132. doi: 10.1186/s13148-021-01116-3 (PMC8240245; doi:10.1186/s13148-021-01116-3)
Supplement: Supplementary file 1 — Additional file 1. Supplementary materials (Figures S1–S8). [file 13148_2021_1116_MOESM1_ESM.pdf]

Oxygen concentration affects *de novo* DNA methylation and transcription in *in vitro* cultured oocytes

Florence Naillat, Heba Saadeh, Joanna Nowacka-Woszuk, Lenka Gahurova, Fatima Santos, Shin-ichi Tomizawa and Gavin Kelsey

Supplementary figure 1: Principal component analysis (PCA) plot for RBBS samples generated from *in vitro* grown (from class 1 to IV) and *in vivo* oocytes (from class 1 to IV).

Supplementary figure 2: Heat map of the informative methylated CGIs in size selected *in vivo* and class IV grown oocytes in 20% O_2 and 5% O_2.

Supplementary figure 3: A) Venn Diagram showing the common CGIs that were affected in GV, 20% O_2 and 5% O_2 oocytes with different level of methylation. B) Correlation regression analysis between GV and IV oocytes, IV *in vivo* oocyte with III *in vivo* oocytes, IV *in vivo* with IV 5% O_2 oocytes and IV *in vivo* with IV 20% O_2 oocytes.

Supplementary figure 4: PCA plot for the RNA-seq from class I to IV *in vivo*, GV, IV 5% O_2 and IV 20% O_2.

Supplementary figure 5: Box whisker plot showing the correlation between identified CGIs and transcription for 20% O_2 and 5%O_2.

Supplementary figure 6: Immunostaining of H3K4me2 and phospho-Foxo3a. A) Oocytes were grown under MAO condition B) Oocytes were grown under LiCl condition. C) GV stained with H3K4me2 and H3K4me3 and phospho-Foxo3 and Fig alpha counterstained with DAPI.

Supplementary figure 7: Drawing of the tree of the replicate samples quantifying CGIs over 0% of methylation for PBAT samples.

Supplementary 8: Quantitation of the methylation level of the 23 igDMRs between MAO, LiCl, MAO+LiCl and normoxia conditions (χ^2 test, $p < 0.05$).

Supplementary table 1: Sequencing output for PBAT libraries.

Supplementary table 2: CGIs overlapping promoter, intragenic and intergenic regions of methylated non-common CGIs after the comparison with the hypermethylated domains defined in *Kdm1a* oocytes and the CGIs methylated >25% in normoxia or 20% O_2 CON, and >10% in MAO, LiCl and MAO+LiCl. PA=promoter, Intra=intragenic and Inter=intergenic.

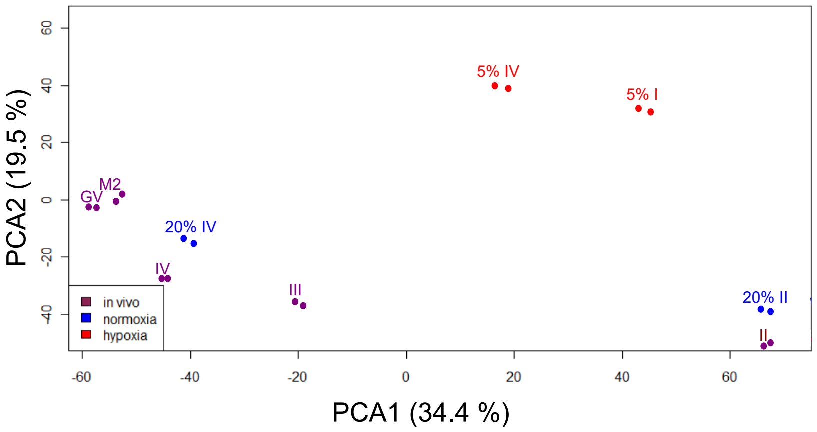

Supplementary figure 1

CGIs methylation n=13 710

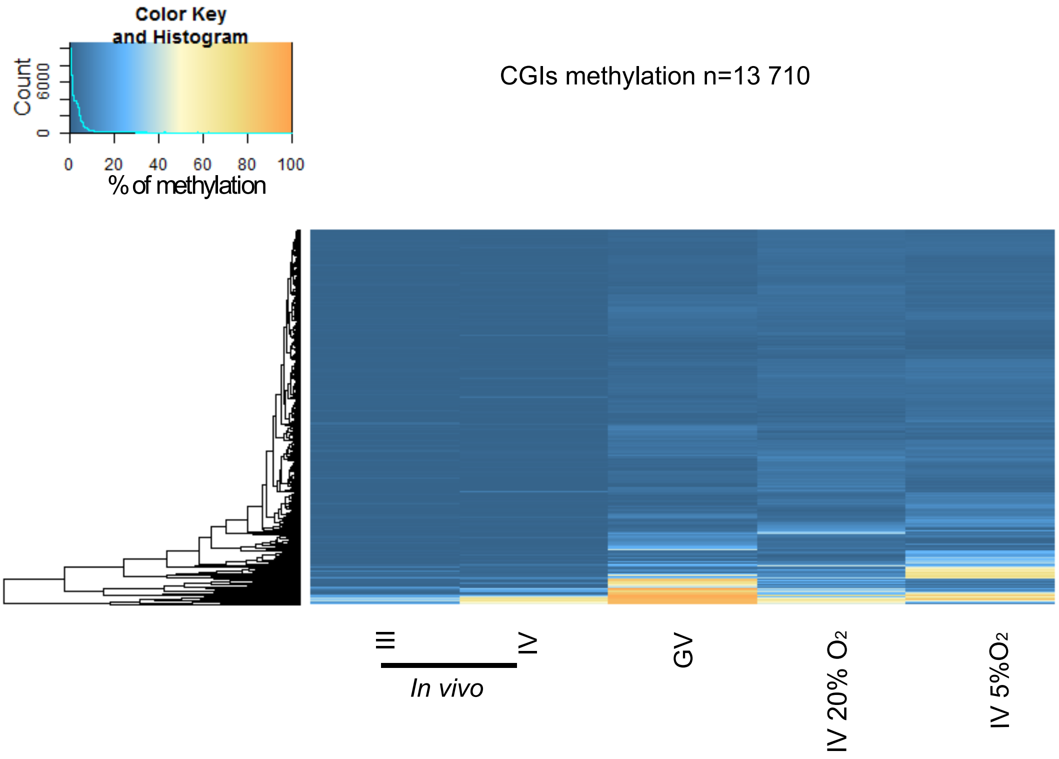

Supplementary figure 2

A

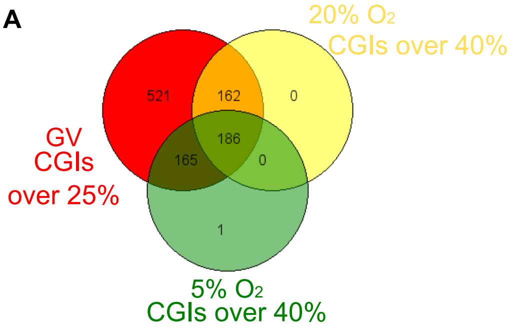

B

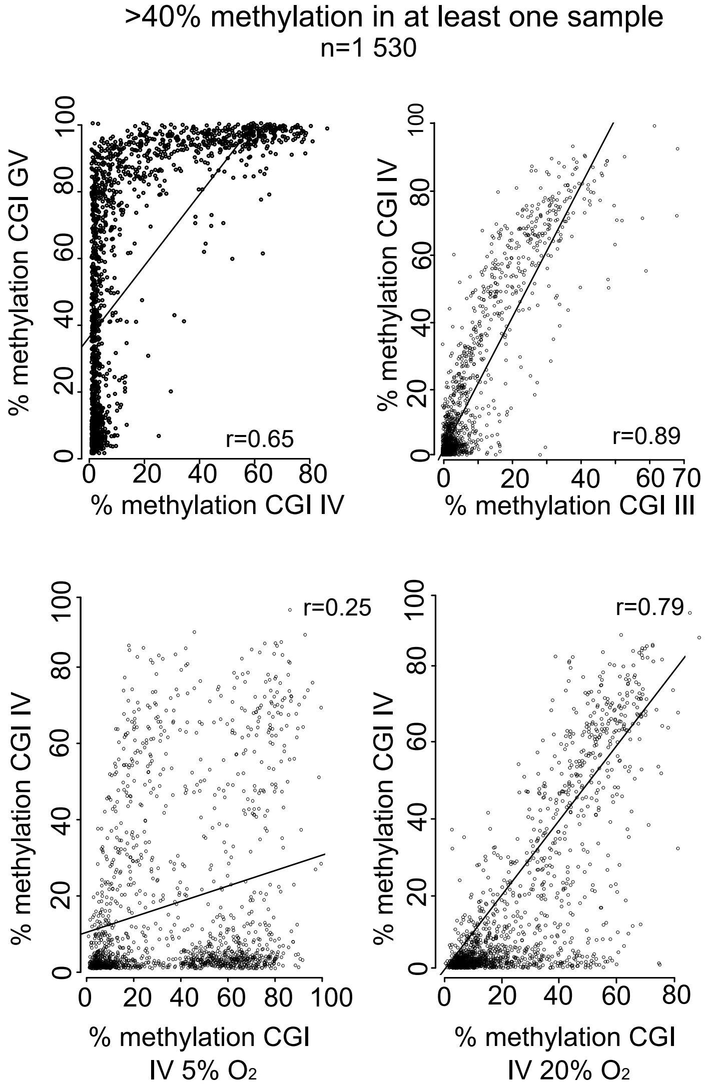

Sppleentary Figure 3

RNA replicates

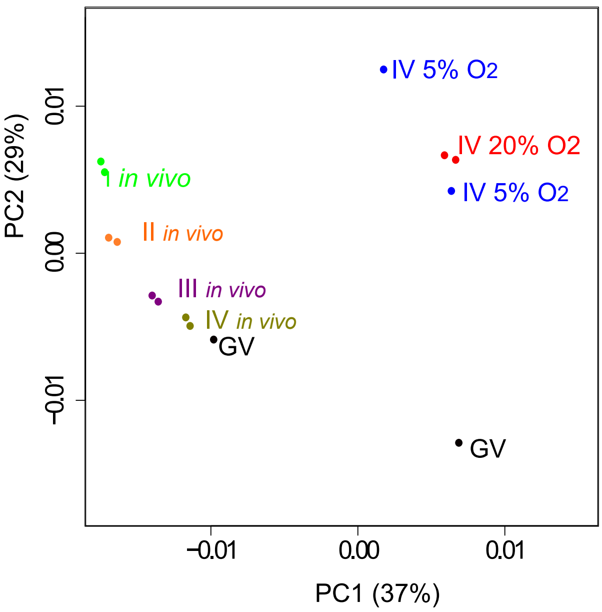

Supplementary figure 4

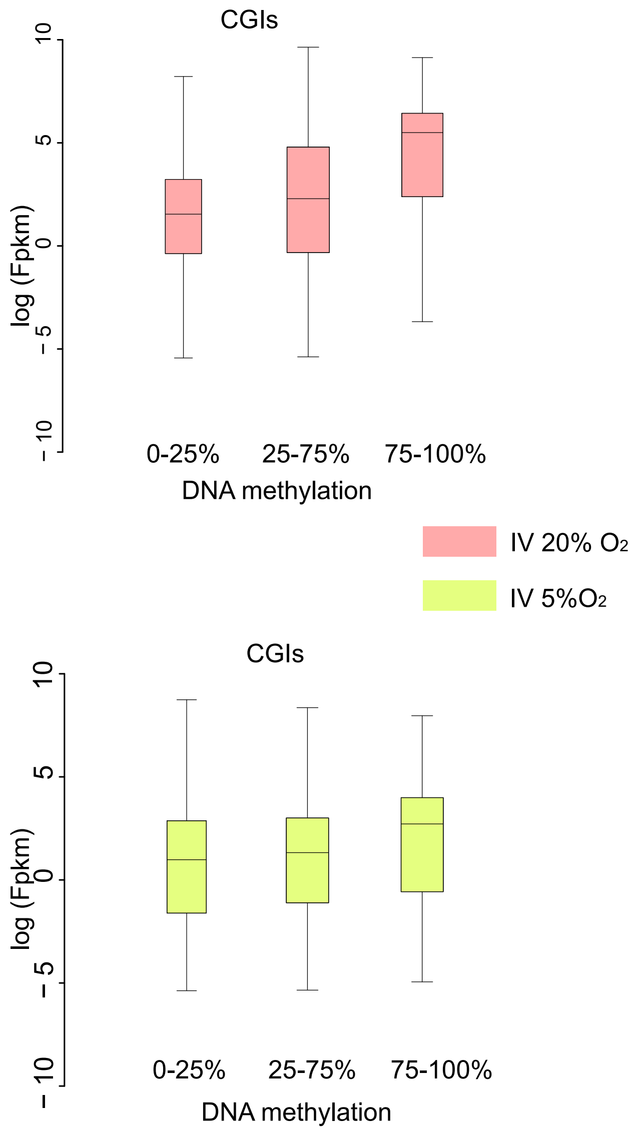

Supplementary figure 5

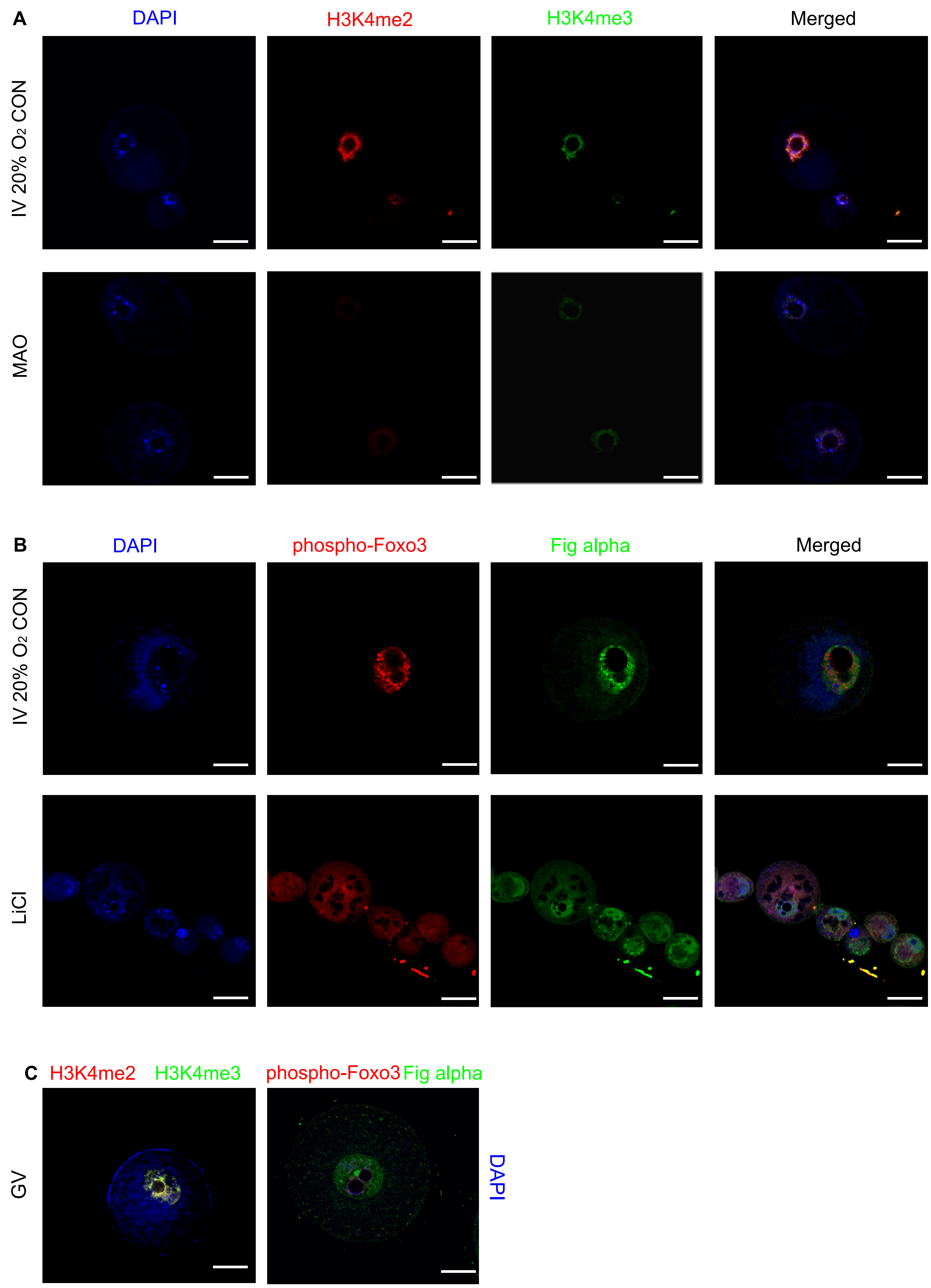

Supplementary figure 6

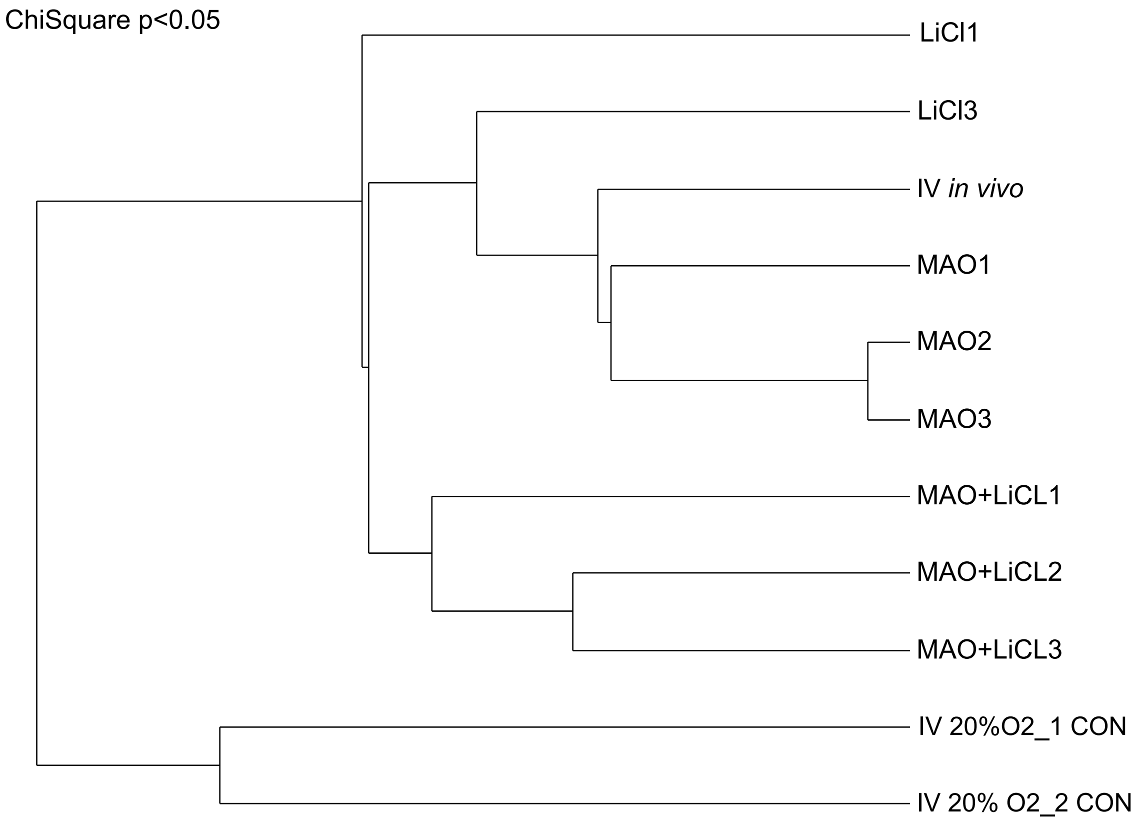

Supplementary figure 7

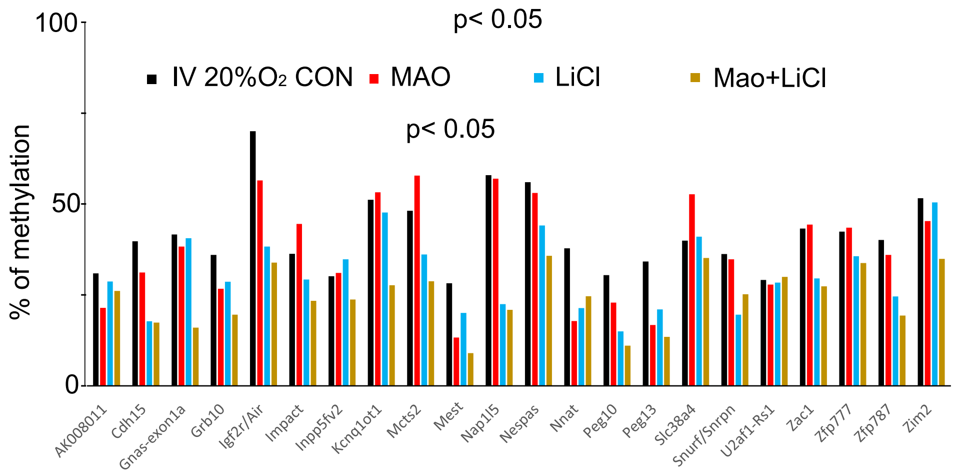

Supplementary figure 8
